# Chronic ischemic ileitis after ileocecal resection and ileocolic anastomosis

**DOI:** 10.1055/a-2078-1024

**Published:** 2023-05-15

**Authors:** Sebastian Vuola, Lucile Héroin, François Habersetzer, Guillaume Philouze, Pierre Mayer

**Affiliations:** 1 Department of Hepatology and Gastroenterology, Pôle Hépato-digestif, Nouvel Hôpital Civil, Hôpitaux Universitaires de Strasbourg (HUS), Strasbourg, France; 2 IHU-Strasbourg (Institut Hospitalo-Universitaire), Strasbourg, France; 3 Inserm U1110, Institute for Viral and Liver Diseases, LabEx HepSYS, University of Strasbourg, Faculty of Medicine, Strasbourg, France; 4 Department of Visceral and Digestive Surgery, Pôle Hépato-digestif, Nouvel Hôpital Civil, HUS, Strasbourg, France

The management of precancerous colonic lesions has radically changed with the progress in endoscopy, especially therapeutic endoscopy. Large or suspected polyps were formerly managed surgically. Endoscopic submucosal dissection has completely changed the management of this kind of lesion, exposing patients to fewer postoperative complications.

We report the case of a 76-year-old women with a history of ileocecal resection with ileocolic anastomosis for benign cecal tumor. A few years later, she presented with chronic abdominal pain, clinically significant weight loss, and recurrent digestive bleeding requiring hospitalization for repeated transfusions 1 . An initial colonoscopy performed during a hemorrhagic period revealed large and longitudinal ulcers on the ileal side of the ileocolic anastomosis. On the anastomosis, there was bleeding ulceration requiring the placement of a clip. Some biopsy samples were taken on the terminal ileum. Histological examination revealed fibrino-granulocytic and necrotic tissue originating from the bottom or the edges of an ulcer. Abdominopelvic computed tomography showed terminal ileitis, without occlusion of a digestive vessel ( Fig. 1 ). Conservative treatment was proposed given the patient’s poor general condition and important cardiovascular comorbidities.

**Fig. 1:**
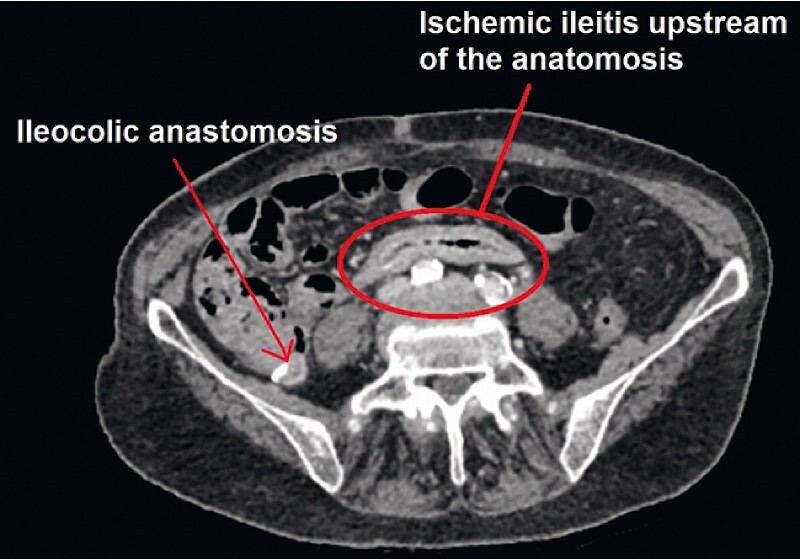
Abdominopelvic computed tomography scan showing thickened aspect of the ileal mucosa in front of the ileocolic anastomosis, corresponding to postsurgical ischemic damage of the ileum.

Unfortunately, symptoms did not improve with medical treatment. A second colonoscopy was performed a few months later ( Video 1 ) and revealed chronic ischemia. The mucosa appeared thin, atrophic, pale, and without vascularization as evidenced by the absence of bleeding during the biopsies ( Fig. 2 , Fig. 3 ). In addition, we found a supracentimetric ulcer associated with a fibrous ileal stenosis 2 3 . After a multidisciplinary board meeting, surgical management with resection of the ischemic ileum and creation of a new anastomosis was planned.

**Video 1** Chronic ileal ischemia following cecal resection with ileocolic anastomosis for huge polyp.


**Fig. 2:**
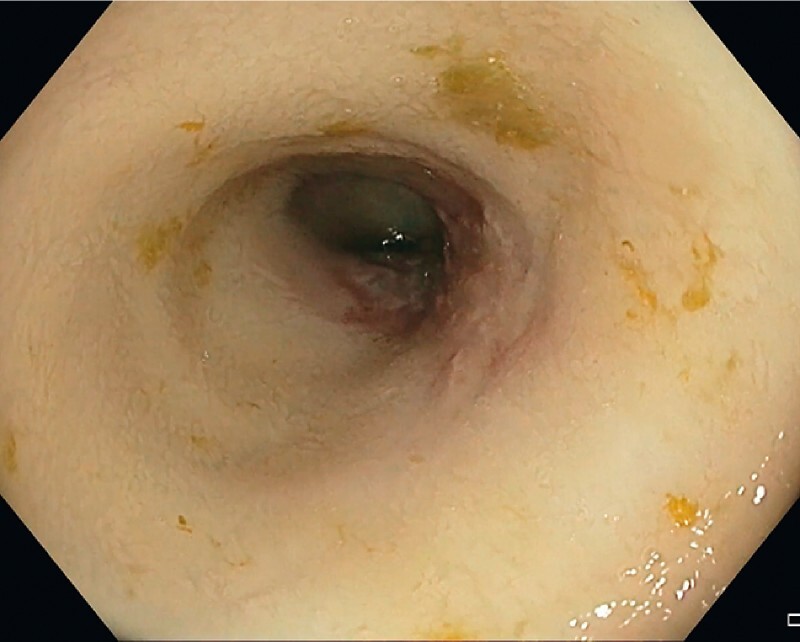
Appearance of chronic ischemia of the terminal ileum, with pale mucosa and no vascularization.

**Fig. 3:**
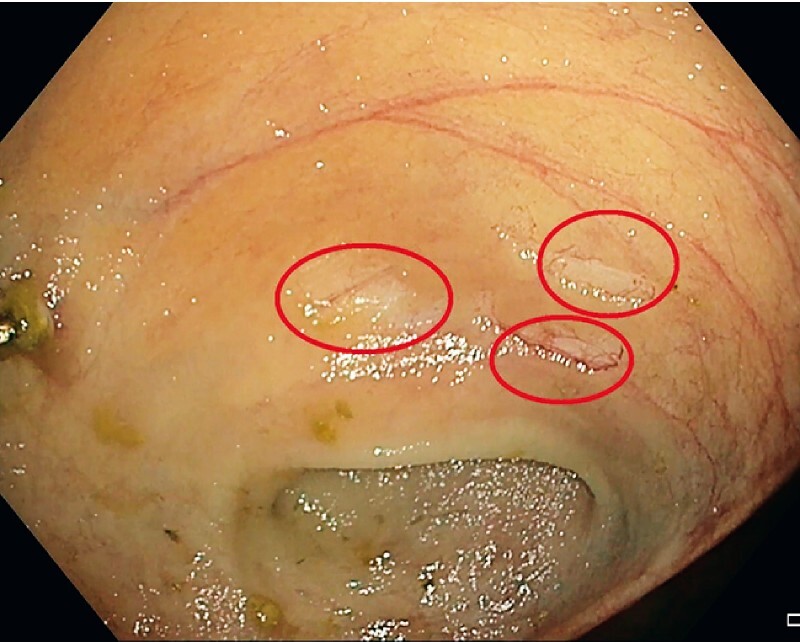
Endoscopic view of the ischemic ileitis, with no bleeding after biopsies (red circles) typical of ischemic phenomena.

This is the first reported description of chronic ischemic ileitis after resection of a noncancerous colonic lesion. It illustrates the importance of endoscopic management of precancerous lesions by endoscopic submucosal dissection, thus avoiding more invasive management and allowing organ preservation.

Endoscopy_UCTN_Code_CCL_1AD_2AF

## Notes

Endoscopy E-Videos

https://eref.thieme.de/e-videos
*E-Videos* is an open access online section of the journal *Endoscopy* , reporting on interesting cases and new techniques in gastroenterological endoscopy. All papers include a high-quality video and are published with a Creative Commons CC-BY license. Endoscopy E-Videos qualify for HINARI discounts and waivers and eligibility is automatically checked during the submission process. We grant 100% waivers to articles whose corresponding authors are based in Group A countries and 50% waivers to those who are based in Group B countries as classified by Research4Life (see: https://www.research4life.org/access/eligibility/ ). This section has its own submission website at https://mc.manuscriptcentral.com/e-videos
